# The assessment and management of sesamoiditis: a focus group study of podiatrists in Aotearoa New Zealand

**DOI:** 10.1186/s13047-023-00628-w

**Published:** 2023-05-16

**Authors:** Preeti Kaur, Matthew R Carroll, Sarah Stewart

**Affiliations:** 1 Department of Podiatry, School of Clinical Sciences, Faculty of Health and Environmental Sciences, Auckland University of Technology, Private Bag 92 006, Auckland, 1142 New Zealand; 2 Active Living and Rehabilitation, Aotearoa New Zealand, Health and Rehabilitation Research Institute, School of Clinical Sciences, Auckland University of Technology, Auckland, New Zealand

**Keywords:** Sesamoiditis, First metatarsophalangeal joint, Qualitative, Focus groups, Podiatry

## Abstract

**Background:**

Sesamoiditis is a common inflammatory condition affecting the sesamoid bones at the plantar aspect of the first metatarsophalangeal joint (1MTPJ). However, there are currently no recommendations or clinical guidelines to support podiatrists in their assessment or management of sesamoiditis. The aim of this study was to explore the views of podiatrists in Aotearoa New Zealand on their approaches to the assessment and management of patients with sesamoiditis.

**Methods:**

This qualitative study included focus group discussions with registered podiatrists. Focus groups took place online via Zoom and were guided by a detailed focus group question schedule. The questions were designed to encourage discussion around assessment approaches used in the diagnosis of sesamoiditis and the treatment tools used to manage patients with sesamoiditis. Focus groups were audio-recorded and transcribed verbatim. Reflexive thematic analysis was used to analyse the data.

**Results:**

A total of 12 registered podiatrists participated in one of three focus groups. Four themes were constructed relating to the assessment of sesamoiditis: (1) obtaining a patient history; (2) recreating patient symptoms; (3) determining contributing biomechanical factors; and (4) ruling out differential diagnoses. Seven themes were constructed relating to the management of sesamoiditis: (1) consideration of patient factors; (2) patient education; (3) cushioning of the sesamoids to allow more comfortable weightbearing of the 1MTPJ; (4) pressure redistribution and offloading of the sesamoids; (5) immobilisation of the 1MTPJ and sesamoids; (6) facilitating efficient sagittal plane motion during gait; (7) referring to other health professionals to find different ways to treat or manage patient symptoms.

**Conclusion:**

Podiatrists in Aotearoa New Zealand demonstrate an analytical approach in the assessment and management of patients with sesamoiditis based on their clinical experience and knowledge of lower limb anatomy. A range of assessment and management techniques are selected based on the practitioners personal preferences, as well as the patient’s social factors, symptomology, and lower limb biomechanics.

**Supplementary Information:**

The online version contains supplementary material available at 10.1186/s13047-023-00628-w.

## Background

Sesamoiditis is a term describing inflammation of the sesamoid bones and surrounding soft tissue structures on the plantar aspect of the first metatarsophalangeal joint (1MTPJ) [1, 2]. Activities involving repetitive stress, such as running, jumping, and forced dorsiflexion of the 1MTPJ, subject the sesamoid bones to greater ground reaction forces, which have been reported to contribute to local irritation and inflammation [1, 3–5]. Consequently, sesamoiditis is more common in athletes, accounting for 2.2% of all foot injuries and 18.3% of all 1MTPJ injuries [6], and is a condition commonly encountered by podiatrists. The prevalence of sesamoiditis in non-athlete populations has not been reported.

The exact pain-related physiological mechanisms involved in the development of sesamoiditis have not been established. Clinically, sesamoiditis is simply described as a sudden or insidious onset of localised or diffuse pain on the plantar aspect of the 1MTPJ [4, 7]. This can result in substantial physical limitations for patients, including difficulty in weightbearing through the 1MTPJ during the propulsive phase of the gait cycle [1, 2, 5, 7, 8]. Although the clinical presentation of sesamoiditis has been described in the existing literature, there is a lack of guidance on the assessment and diagnosis of sesamoiditis.

Non-surgical management options that have been documented for sesamoiditis include immobilisation techniques via splints, shoes, moonboots, and special orthoses, as well as the use of anti-inflammatory drugs, rest, activity modification, cortisone injections, and radial shockwave therapy [1, 2, 5, 9]. However, the effectiveness of various conservative interventions for sesamoiditis has not been established, and there are currently no published recommendations or guidelines for their use in clinical practice.

An understanding of the current assessment and management practices used by podiatrists is an important step in developing recommendations that can aid in the podiatric assessment and treatment of sesamoiditis. The aim of this study was therefore to explore the views of podiatrists in Aotearoa New Zealand on their approaches to assessing and treating sesamoiditis.

## Methods

### Design

A qualitative focus group study was conducted. The design and conduct of the study were influenced by interpretivism, in which reality is observed subjectively [10]. An inductive approach was used to collect and analyse data, meaning that the knowledge produced solely reflected the experiences and perceptions shared by the study participants.

### Participants

Participants were included if they were registered podiatrists in Aotearoa New Zealand, had at least five years of clinical experience, and had a predominantly (~ 60%) biomechanical/musculoskeletal clinical caseload. These criteria were chosen to ensure targeted recruitment of participants who had sufficient experiences in the assessment and management of musculoskeletal-based pathology in order to generate high-quality dialogue that specifically addressed the research aim. Probability sampling was used to recruit participants via email invitations through the Podiatrists Board of New Zealand and the New Zealand Accredited Sports Podiatry Group. A sample size of 12 podiatrists (three focus groups of four participants each) was determined by the concept of Information Power (as opposed to data saturation). Our high level of information power was achieved through the two highly specific predefined research aims, the inclusion of only participants with predominantly biomechanical/musculoskeletal caseloads, the expected high quality of dialogue, the use of a piloted focus group schedule to guide the discussion, and the planned in-depth analysis approach [11]. This study was approved by the Auckland University of Technology Ethics Committee (AUTEC 22/72) and all participants provided written informed consent prior to data collection.

### Data collection

Data was collected in three focus group discussions held online via Zoom between July and August 2022. The focus group approach was chosen to allow exploration of the collective perspectives, experiences and opinions of podiatrists through lively discussion and production of rich and meaningful data [12]. A detailed focus group question schedule (Supplementary File 1) was developed by the research team and piloted with three podiatrists (outside of the study participants) to further refine the questions and relevance of the language and wording. The questions posed to participants were intended to encourage discussion of assessment approaches used in both the assessment and the diagnosis of sesamoiditis and the treatment tools used in the management of patients with sesamoiditis. All focus groups were facilitated by a single researcher (PK) whose role was to guide discussion using prompts and follow-up questions to stimulate discussion. The question schedule was adjusted over the data collection period and new questions were added to gain a deeper understanding of specific perceptions. All focus groups were audio recorded and transcribed verbatim by an external transcription service.

### Data analysis

Data analysis was performed simultaneously with data collection using reflexive thematic analysis [13, 14]. This approach was deemed most appropriate as it allows the researchers’ engagement with the data to be guided by their research question and theoretical background and values [15]. Reflexive thematic analysis allows the generation of new meaning by producing patterns (themes) within the data. This included initial familiarisation with the data by reading the transcripts and listening to the audio recordings multiple times [15]. Initial latent and sematic codes were developed by a single researcher (PK) using NVivo software, with each code representing a label for a distinct piece of information that was relevant to the research aim. Coding was undertaken separately for assessment and management approaches to sesamoiditis. Multiple iterations of coding were undertaken and sense-checked by the other researchers (SS, MC). Prototype themes were then constructed and refined by the research team to ensure that each theme contained a strong central organising concept and that the themes were conceptually rich and distinct [16]. The final themes were then reviewed, defined, and named by the research team. Illustrative quotes were chosen to support each theme and mind maps were created to provide a visual representation of how the themes fit together.

## Results

### Participant characteristics

The characteristics of the 12 podiatrists included in the study are presented in Table 1. The number of years in podiatric practice ranged from eight to 44. Most participants received their initial education in Aotearoa New Zealand and two thirds went on to obtain postgraduate qualifications. All participants practiced in the private health sector.

**Table 1:** Participant characteristics

**ID**	**Sex**	**Focus group**	**Years in practice**	**Country of training**	**Qualification**	**Countries practiced in**	**Current region of practice**	**Private or public**
**P01**	Female	#1	44	United Kingdom	Postgraduate	Australia, New Zealand, United Kingdom	South Island	Private
**P02**	Female	#1	15	New Zealand	Postgraduate	Australia, New Zealand, United Kingdom	North Island	Private
**P03**	Male	#1	13	New Zealand	Postgraduate	New Zealand	North Island	Private
**P04**	Male	#1	20	New Zealand	Undergraduate	New Zealand	North Island	Private
**P05**	Male	#2	14	New Zealand	Postgraduate	New Zealand	North Island	Private
**P06**	Male	#2	08	New Zealand	Postgraduate	New Zealand	North Island	Private
**P07**	Female	#2	14	New Zealand	Postgraduate	New Zealand	North Island	Private
**P08**	Female	#2	10	New Zealand	Postgraduate	New Zealand	North Island	Private
**P09**	Male	#3	35	New Zealand	Undergraduate	New Zealand	North Island	Private
**P10**	Female	#3	09	New Zealand	Postgraduate	Australia, New Zealand	North Island	Private
**P11**	Male	#3	25	New Zealand	Undergraduate	New Zealand	South Island	Private
**P12**	Male	#3	18	New Zealand	Postgraduate	Australia, New Zealand	South Island	Private

### Assessment of sesamoiditis

**Fig. 1:**
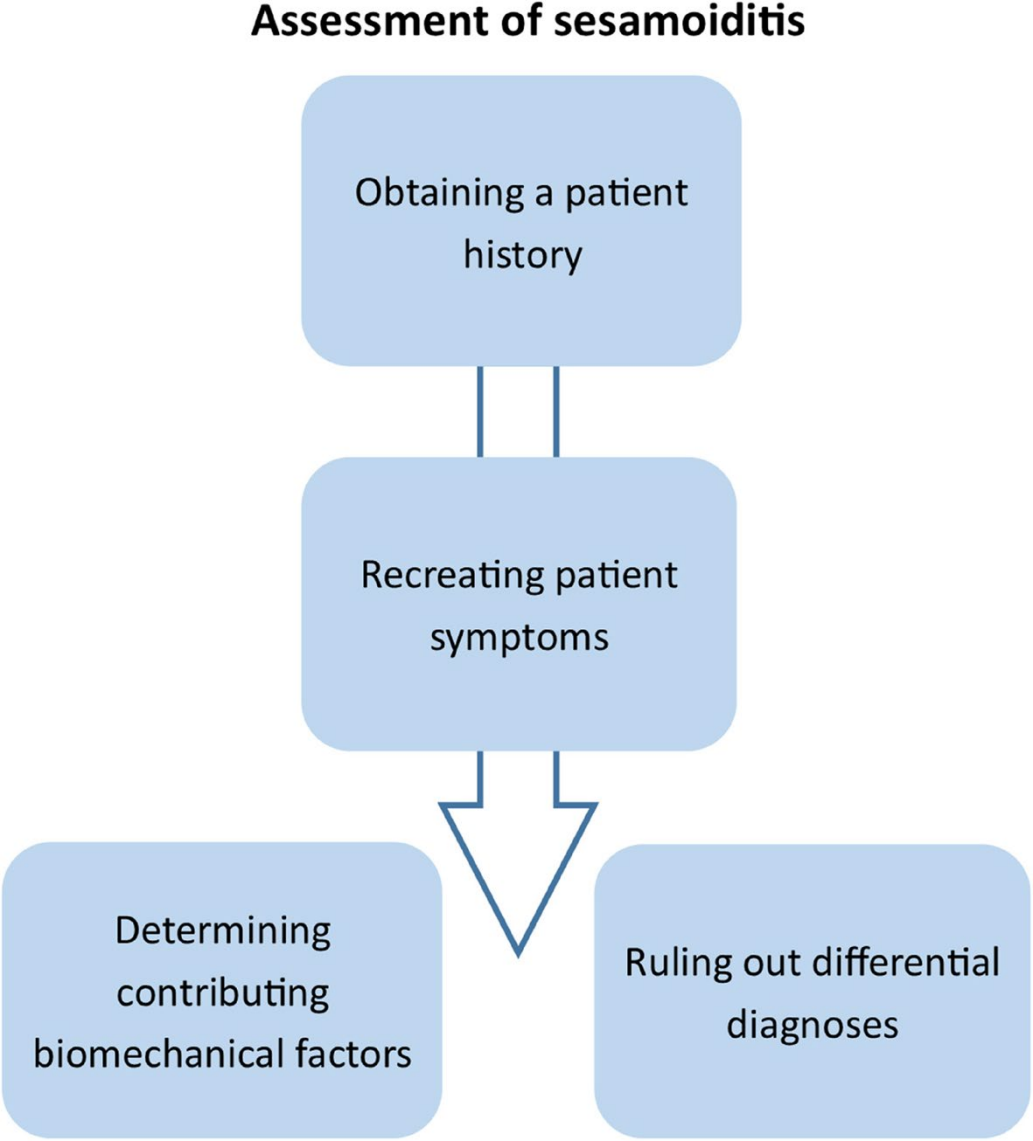
Themes representing podiatrists’ approach to the assessment of sesamoiditis

Four major themes were constructed relating to assessment and diagnosis of sesamoiditis: (1) obtaining a patient history; (2) recreating patient symptoms; (3) determining contributing biomechanical factors; and (4) ruling out differential diagnoses (Fig. 1).

#### Theme 1. Obtaining a patient history

Obtaining information about patient-reported symptoms was described as important by most practitioners:

*“I probably try to get a lot of information from my subjective assessment which is then gonna guide to the approximate region that the person is talking about, try to work out what kind of tissues are involved” (P03).*

In addition to determining whether the symptoms represented acute or chronic pathology, practitioners also focused on understanding the mechanism of injury:

*“I get them to, if they can, replay or show me the mechanism” (P05)*.

Mechanisms subjecting the 1MTPJ and sesamoids to high loads and stress were considered useful in establishing a diagnosis. Many podiatrist also noted a link between sesamoid injuries and sports involving firm surfaces, high propulsion, rotational movements, and/or repetitive movement and impact:

*“Early season rugby [or] football training where the ground is still hard, there seems to be a little bit of an influx where you might get quite a few people in with pain in that sesamoid area” (P02).*

#### Theme 2. Recreating patient symptoms

Direct palpation of the sesamoids was the most common method used to elicit symptoms:

*“I normally just push on the sesamoid bone and go, oh, yeah” (P06)*.

Some podiatrists also described performing a passive axial compression test which allowed them to.

*“ … get in there and give it a good wobble” (P08)*.

This test involved placing the hallux in a maximal dorsiflexed position (causing distal migration of the sesamoids), before applying proximal compression to the sesamoids to stabilise them. The hallux is then plantarflexed and if symptoms are produced this indicates a positive test.

Dynamic tests such as resisted muscle testing, hopping tests, and heel raises were also commonly used to recreate symptoms by testing the sesamoid pully function and simulating the propulsive phase of gait:

*“I like the calf raises, seeing how much they can tolerate, whether they can only do double leg or whether they can do single leg.” (P06)*.

#### Theme 3. Determining contributing biomechanical factors

After establishing the diagnosis of sesamoiditis, additional assessments aimed at determining contributing biomechanical factors made up a large part of the initial appointment. Some assessments were specific to the sesamoid area and included testing of 1MTPJ range of motion and first ray position (relative to the other metatarsal heads) to gain a better understanding of the windlass mechanism and loading during propulsion. The degree of transverse sesamoid translation and potential subluxation as a cause of local inflammation was also considered:

*“Sometimes, in my opinion, that lateral sesamoid tends to be slightly more deviated so I like to give it a bit of a push from lateral and medial to see if that exacerbates any pain” (P10).*

Practitioners frequently reported seeking further information about the rearfoot to forefoot relationship by assessing rearfoot position and overall foot posture:

*“If they have a particular foot type where the way that they walk is really overloading that particular area … working around that and thinking more of the long term and thinking more preventatively as well so it doesn’t become a recurring issue for them” (P02).*

Some podiatrists also considered assessment of the entire lower limb, including leg length discrepancies and hip positions in order to determine whether more proximal factors could be contributing:

*“I start at the pathology site of concern and then work my way in reverse trying to look for patterns that could explain why this is occurring” (P04)*.

In addition to these static non-weightbearing assessments, podiatrists also sought to improve understanding of the patients walking or running mechanics and how these may have contributed to development of sesamoiditis:

*“ … depending on what activity they do, say, they’re doing sport or something, I probably go and get them having a bit of a run, just to see what aggravated it” (P07)*.

Practitioners who had plantar pressure systems in their practices also found these beneficial:

*“Weight distribution point of view, with a in shoe pressure or pressure plate is actually a good assessment tool in terms of understanding loading patterns through that joint.” (P12).*

Finally, biomechanical-based assessments also extended to the patients’ footwear to gain an understanding of their role in the development of sesamoiditis:

*“I quite like looking at the insole of the shoe too, pulling that out and having a look and see if there’s any obvious wear pattern can sometimes be a give-away” (P04)*.

Assessment of sprig placement on rugby and football boots, particularly if positioned beneath the 1MTPJ, was also of interest. Shoes with poor structure and a minimalistic sole style were often reported as causative factors due to lack of cushioning and increased sesamoid loading, as was fit of the shoe:

*“Sometimes people buy a boot that’s too small or too big and that really affects [how] that metatarsophalangeal joint extends and flexes” (P05)*.

#### Theme 4. Ruling out differential diagnoses

Podiatric tests were undertaken by the podiatrists in order to rule out other sesamoid pathology, including fractures, bipartite sesamoids, and avascular necrosis. Pathology of neighbouring structures, including ligaments, joint capsules, plantar plates, distal insertions of the flexor hallux longus and brevis tendons and the insertion of the abductor hallucis and plantar fascia were also considered. These assessments were based on utilising detailed knowledge of local anatomy:

*“I think we forget sometimes that we actually probably know the foot anatomy better than anyone else” (P08)*.

Direct referral for imaging investigations, including ultrasound and plain radiography were sometimes used to further assist in ruling out differential diagnoses:

*“I don’t tend to use much; I think mostly it’s a clinical thing. If I’m suspecting a fracture or bipartite sesamoid or something like that, potentially an x-ray” (P03)*.

In some situations, CT and MRI were also considered valuable, however, referral to these services within the Aotearoa New Zealand funded health system pathways were challenging for podiatrists and their patients. Under the Accident Compensation Corporation (ACC) scheme podiatrists can refer for plain radiography and ultrasound, but any further funded imaging requires a referral through a sports physician or orthopaedic surgeon, which places another step and time delay in the referral pathway:

*“I would like to be able to at some stage, refer for MRI or CT, because I think we know enough to do it but … it’d be great to see in greater detail what’s happening in a specific joint” (P05).*

Practitioners in the current study also reported referring to other health professionals to conduct assessments that were beyond their scope of practice, including to general practitioners for blood tests to rule out underlying systemic causes of sesamoid inflammation and to sports physicians or orthopaedic specialists when imaging outside of plain radiography and ultrasound were required.

### Management of sesamoiditis

**Fig. 2:**
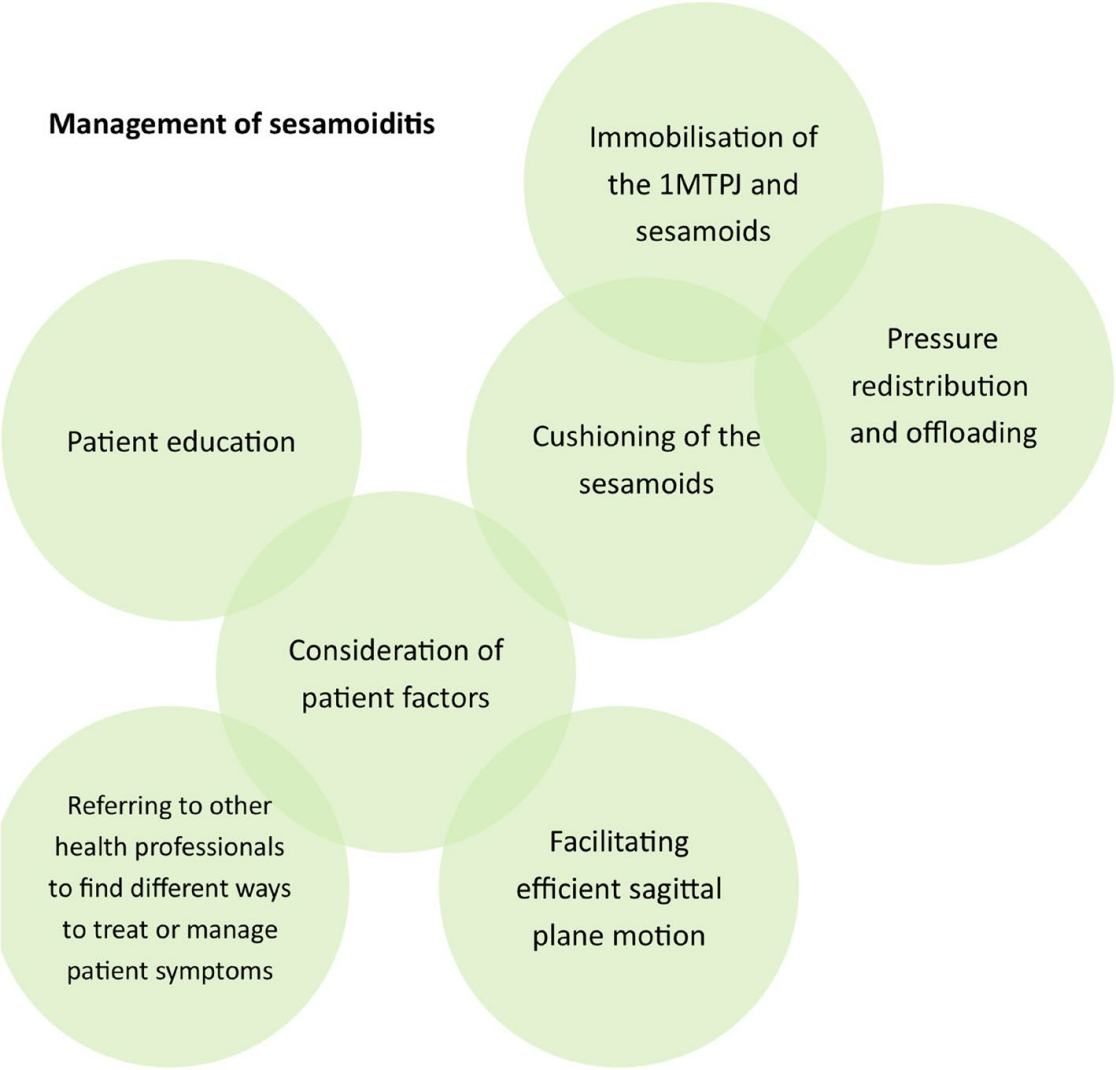
Themes representing podiatrists’ approach to the management of sesamoiditis

Seven major themes were constructed relating to the management of sesamoiditis. These were: (1) consideration of patient factors; (2) patient education; (3) cushioning of the sesamoids to allow more comfortable weightbearing of the 1MTPJ; (4) pressure redistribution and offloading of the sesamoids; (5) immobilisation of the 1MTPJ and sesamoids; (6) facilitating efficient sagittal plane motion during gait; and (7) referring to other health professionals to find different ways to treat or manage patient symptoms (Fig. 2).

#### Theme 1. Consideration of patient factors

The podiatrists in the focus groups agreed that there was no single effective approach to treating sesamoiditis:

*“Something obviously will work for someone and it doesn’t work for someone else so, … creativity is key here … is it gonna fit into the shoe? Is it really realistic workwise? How long are they gonna be weight bearing on it from day-to-day? Is it durable? All of these variables you have to take into consideration when you are creating a management plan” (P10).*

Several podiatrists also spoke about the patients’ financial situation, particularly when prescribing orthotics or recommending footwear, both of which can be expensive investments.

*“I’ll start really simple, like … just modifying the insole/sock liner if it’s on the lower budget end. If we’ve got a little bit more money to work with then we’ll do a prefab or a custom orthotic” (P06).*

The cost-barrier was particularly evident for podiatrists working in lower socioeconomic rural areas where many podiatrists also struggled with access to suitable and appropriate footwear options.

#### Theme 2. Patient education

Improving patient understanding through clear education and communication was considered essential to an effective management programme:

*“I’m gonna spend that time educating them about what I think it is and how I think we’re gonna manage it and also what I think my goal posts are gonna be” (P09)*.

Reduction of pain and inflammation was a central focus of patient education. For many podiatrists, education also encompassed training and load management principles involving activity modification until symptoms reduced. RICE (rest, ice, compression, elevation) was recommended for acute stages of sesamoiditis, but most podiatrists discussed pharmacological management with their patients. As podiatrists in Aotearoa New Zealand are limited by an absence of prescription rights, this involved recommending over-the-counter medications, or referral to a pharmacist or GP to provide prescriptions:

*“I’m not a pharmacist so I don’t wanna get in trouble, but I certainly do recommend a course, just to take the edge off” (P10)*.

Some practitioners highlighted the importance of communication and education when patients’ expectations may have been less realistic:

*“I am really brutal about women’s footwear, and I basically refuse to treat anything which is gonna make my treatment compromised so I do sit down with people and have a pretty hard talk about how we’re gonna make it work” (P09).*

#### Theme 3. Cushioning of the sesamoids to allow more comfortable weightbearing of the 1MTPJ

Cushioning of the sesamoids was considered during initial stages to allow more comfortable weightbearing through the 1MTPJ. This was achieved through footwear and/or orthoses.

Providing advice to patients about avoiding thinner soled shoes (i.e., racing flats) and option for trainer-type footwear with adequate cushioning was commonly reported:

*“I’d say rocker sole, really thick cushion shoes and then gel forefoot cushion shoes” (P06)*.

Likewise, avoidance of high-heeled shoes, which not only lack cushioning, but also increase forefoot load was also important:

*“I think it kind of goes without saying … it’s obviously avoiding high heel shoes. Probably a really important point to make in your management” (P08)*.

Further cushioning via orthotic prescription was achieved via the addition of softer materials:

*“I’ll use a softer based orthotic. We usually use a cushion foot bionic and pretty much some kind of first MTP pad, often double layered and, again, well bevelled” (P09)*.

#### Theme 4. Pressure redistribution and offloading of the sesamoids

The use of a moonboot to offload the sesamoids was described in situations where the patient had difficulty weight-bearing, where a fracture was suspected, or where severe or acute inflammation of the sesamoids was evident:

*“ … in a moon boot for a week … that generally works pretty well to help settle things down” (P02)*.

Padding, using either semi-compressed felt or poron, which was applied to the liner of the moon boot or shoe, or directly to the patients feet, was also used as a temporary offloading technique:

*“Patient turns up and there’s no way you’re gonna get a prefab or something into a [dress] shoe, so just a simple offloading principle as soon as you can, if there’s no other option. Certainly it’s only a very temporary method because most of that padding will break down” (P11).*

Orthoses were used for more permanent offloading of the sesamoid area including plantar ‘U’ covers, first ray cut-outs, and metatarsal domes. Reverse Morton’s were often combined with arch fill to redistribute pressure from the sesamoids to the arch area:

*“2 to 5 s [reverse Morton’s] just seem to work. I know they shouldn’t but for some reason they seem to work quite well” (P05)*.

However, other podiatrists were concerned that this modification would load the area further:

*“I’d never think to do a reverse Morton’s because I’d be too concerned with plantar flexing that first ray, that you would get more pain” (P08)*.

Materials were also discussed as an important consideration when constructing orthotic modifications for offloading the sesamoids:

*“Definitely does depend on the foot that we’re seeing. The majority of the time, I’d do a firm density EVA that’s relatively thick.” (P10)*.

Finally, many podiatrists also discussed sprig removal in football boots as a mechanism to offload pressures at the plantar 1MTPJ:

*“Another thing I often do … with football boots I typically take that cleat out under that first metatarsal phalangeal joint as well” (P08)*.

#### Theme 5. Immobilising the 1MTPJ and sesamoids

Podiatrists reported using strapping tape as a technique to immobilise and resist movement at the 1MTPJ during the propulsive phase of gait and to determine the potential effectiveness of orthotic therapy:

*“I do a bit of strapping tape as well to reduce the motion of the first MTPJ and if that feels really good then I’ll implement that into an orthotic with a Morton’s extension” (P10).*

A Morton’s extension was the preferred orthotic modification by many podiatrists to immobilise the 1MTPJ and prevent loading through the sesamoid pivot. However, as the Morton’s extension follows the opposite concept to a reverse Morton’s (i.e., immobilisation vs. offloading), this modification was sometimes used with caution and viewed as a temporary immobilisation strategy:

*“If we restrict one joint and typically what you do with the Morton’s extension is we don’t open up a can of worms for other injuries or pathologies” (P08)*.

Other podiatrists described changing the density of the Morton’s extension (usually a hard plastic) to a softer material over time as the sesamoid inflammation reduced or covering the modification with a softer plantar top cover.

Stiff carbon fibre plates were used when a greater degree of immobilisation was required:

*“I’ve got [rugby] players that are actually still playing in them all season even though [the ground has] got really soft and they’ve just been using it cos it gives them some stiffness through scrummaging and changing their load” (P05).*

Carbon fibre material was sometimes combined with orthotic therapy. However, it was used with caution by some due to patient acceptability and comfort, particularly when combined with orthotic therapy:

*“I don’t find there’s enough room for an orthoses either prefab or custom and a carbon plate unless you’ve got something like a work boot scenario” (P12)*.

#### Theme 6: Facilitating efficient sagittal plane motion during gait

Facilitating a more efficient heel-to-toe gait pattern was a key theory influencing many management techniques for sesamoiditis:

*“I’m trying to enhance sagittal plane movement to try and move them through that area more quickly or change loading through the forefoot to redistribute load more evenly away from that area.” (P03).*

Podiatrists often recommended shoes with rocker soles to facilitate propulsion through the 1MTPJ:

*“That great little rocker through there makes a massive difference to unloading that joint because minimal extension takes place in the toe when they’re walking” (P11)*.

In addition to footwear, inclusion of strengthening exercises targeting the flexor hallucis longus muscle and other intrinsic foot muscle was believed to encourage more efficient loading through the 1MTPJ during propulsion. Other podiatrists described using towel grip exercises, resisted flexion and spikey ball exercises to increase strength in the intrinsic foot musculature.

Long term orthotic modifications were also focused on improving more efficient loading through the 1MTPJ. Customised plantar metatarsal pads (PMP), kinetic wedges, and metatarsal bars were forefoot orthotic modifications used by participants to help sagittal plane motion:

*“ … using something like a PMP to try and spread load across that part of the foot so that I can allow them to carry continuously through or efficiently through the sagittal plane without excessively placing load on the first MPJ” (P03).*

The podiatrists also recognised the importance of rearfoot posting to facilitate sagittal plane motion while also preventing excessive loading of the medial forefoot.

#### Theme 7. Referring to other health professionals to find different ways to treat or manage patient symptoms

Referral to wider interdisciplinary team members for further management was discussed among the participants as an approach for patients whose symptoms did not reduce with podiatry care.

Referral practices were dependent on specific time frames for many podiatrists:

*“I feel like sesamoiditis, you get a fairly quick indication of whether your treatment plans’ working but maybe four to six weeks if it wasn’t working, I’d go and refer on” (P07)*.

Some practitioners referred earlier to factor in the waiting time to see a sports physician or orthopaedic surgeon.

It was clear among the participants that there was hesitancy in referring for more invasive therapies due to side effects and low success rates and were often only considered as last resorts:

*“If we haven’t got the skills to do it ourselves, then there’s really only the surgeon and, as I say, I haven’t found surgery to be successful for sesamoiditis but maybe I haven’t seen enough” (P01).*

## Discussion

This study provides new insight into the assessment and management practices used by podiatrists in Aotearoa New Zealand for patients with sesamoiditis. The results show that podiatrists perform a range of investigations to diagnose and guide the management of sesamoiditis. They also employ a number of management strategies tailored to the individual.

A thorough approach to assessing patients with sesamoiditis was reported by podiatrists in the current study and was driven by knowledge of lower limb anatomy and function. The assessments were used to make a diagnosis, but also to identify biomechanical factors that contributed to the development of sesamoiditis, which could then be considered in the management plan. Assessment techniques included both subjective questioning and objective examination, with podiatrists recognising the functional interdependence between the sesamoid complex and other regions of the lower limb [17]. The reported reliability and validity of foot and ankle assessments varies widely [18] and has not been specifically studied in people with sesamoiditis. Further research into the measurement properties of commonly used lower limb assessments, including diagnostic accuracy, is warranted and may refine the choice of assessments used by podiatrists [17]. Participants also recognised the value of referral for additional assessments available outside of their scope, including advanced imaging, to support diagnosis. Plain radiography and magnetic resonance imaging are currently the gold standard for imaging of sesamoiditis, followed by computed tomography as the third modality of choice [5, 19–23]. Podiatrists in Aotearoa New Zealand can only refer for plain radiography and ultrasound imaging of the lower limb. As a result of an inability to directly refer for other imaging assessments, patients may face delays in diagnosis and the podiatrist's ability to provide timely and effective care.

Patient education and clear communication regarding diagnosis and management were seen as key concepts to improve patients’ understanding of their condition. Education included training, load management principles and activity modification until symptoms reduced. Reducing pain and inflammation was a key focus of patient education. Although RICE therapy is recommended for acute stages of sesamoiditis, most podiatrists discussed pharmacological management with their patients. Because podiatrists in Aotearoa New Zealand are constrained by an absence of prescription rights, management is limited to recommending over-the-counter medicines, or referral to a pharmacist or general practitioner to provide prescriptions. As with referral for advanced imaging, direct referrals for the most effective pharmacological treatment strategies are not possible, and patients often face the additional time and financial cost associated with secondary referrals, limiting the podiatrist's ability to provide timely and effective care.

The treatment approaches described varied between practitioners and depended on the podiatrist’s personal preferences and the patient’s social factors, symptomology, and biomechanical findings. Prescribing foot orthoses and footwear were central components of practitioner management and served not only to cushion and redistribute loads from the sesamoid bones, but also to immobilise or facilitate efficient sagittal plane motion through the 1MTPJ. The clinical reasoning described by practitioners underscores the shift from the Root theories’ focus on restoring the foot’s “ideal alignment” to concepts informed by the Tissue Stress Model [24] and the Preferred Movement Pathway [25]. Consistent with a recent report on the foot orthoses prescription habits among New Zealand podiatrists, [26] a combination of prefabricated and custom foot orthoses were prescribed, dependent on the patient’s individual presentation and management goals.

Podiatrists in the current study also expressed reluctance to refer for surgical intervention because of a perceived low success rate. These views reflect the varied postoperative outcomes reported in the literature [3, 23, 27–29]. Although the surgical management of sesamoiditis is not without risk and is not considered to be strongly evidence-based or protocol-driven [28], a meticulous surgical technique and a postoperative rehabilitation program can lead to successful postoperative outcomes and a reduced risk of complications [27].

This study should be considered in light of some limitations. First, the focus groups were facilitated by an experienced sports podiatrist, who also contributed to the analysis and interpretation of the results, which may have introduced personal bias. However, reflective thematic analysis recognises that research occurs due to the subjectivity of the researcher and does not attempt to eliminate it [15]. In addition, the experience of the focus group facilitator, who had a sound understanding of podiatric concepts and terminology, ensured that the discussions were focused and specific to the research aim. Second, this study only included podiatrists practicing in Aotearoa New Zealand, and their experiences may not reflect other geographical locations, cultural factors, or healthcare systems. Further research exploring the experiences of podiatrists in wider geographical regions would improve generalisability. Finally, although the targeted recruitment strategy ensured participants had sufficient experience in assessing and manging musculoskeletal pathology, including sesamoiditis, podiatrists who have predominantly older adult caseloads were not included and they may have different experiences of assessing and managing sesamoid pathology resulting from natural deterioration of joint function.

The findings from this study have highlighted diverse and distinct management strategies ranging from cushioning, pressure redistribution, and immobilisation, to enhancement of sagittal plane motion. However, further work is required to determine how assessment outcomes can be used to inform the most optimal management strategy, including when referrals should be instigated, and whether adjunct modalities used successfully in other chronic pain conditions including plantar heel pain and Achilles tendinopathy may also play a role (e.g., electrophysical agents including extracorporeal shockwave therapy, and iontophoresis) [30, 31]. Prospective studies, and ideally, randomised controlled trials, are needed to evaluate the effectiveness of different podiatric interventions for sesamoiditis, specifically cushioning, offloading and immobilisation techniques that can be tailored to the individual. Coupled with the data from this study, this information can then be used to develop evidence-based guidelines and clinical practice recommendations that podiatrists can use when managing people with sesamoiditis.

In conclusion, despite the absence of guidelines and recommendations, the data from this study demonstrated that podiatrists in Aotearoa New Zealand use an analytical approach in assessing and managing patients with sesamoiditis, based on their clinical experiences and anatomical knowledge of the lower limb. A range of assessment and management techniques are selected based on the individuals social factors, symptomology, and lower limb biomechanics.

## Supplementary Information

**Additional file 1: Supplementary File 1. **Focus group question schedule.

## Data Availability

The transcripts that support the findings of this study are not available publicly due to participant confidentiality.

## Abbreviations

1MTPJ: First metatarsophalangeal joint
ACC: Accident Compensation Corporation
