# Supplementary material for: The assessment and management of sesamoiditis: a focus group study of podiatrists in Aotearoa New Zealand
Source: J Foot Ankle Res. 2023 May 16;16:29. doi: 10.1186/s13047-023-00628-w (PMC10186644; doi:10.1186/s13047-023-00628-w)
Supplement: Supplementary file 1 — Additional file 1: Supplementary File 1. Focus group question schedule. [file 13047_2023_628_MOESM1_ESM.docx]

**SUPPLEMENTARY FILES**

**Supplementary File 1.** Focus group question schedule

*The following focus group schedule was used by the facilitator as a guide only. Possible prompts were suggested below and were used, as required, to facilitate the discussion.*

**[Facilitator’s introduction to focus group]:**

Welcome to you all. Firstly, I’d like to thank you for your time and your interest in my research. I’m Preeti, a registered podiatrist, and will be the facilitator for this discussion. I have been in private practice now for seven years and am very much interested in forefoot injuries with a keen interest in sesamoid injuries. The goal of this discussion is to share your thoughts and experiences around the assessment and management of sesamoiditis. Just to remind you – our discussion today will be recorded, but all the data will be de-identified to ensure that you are not identified personally in any report/publication arising from the research.

Some ground rules I’d like to mention before we start:

- There are no right or wrong answers. I encourage you to speak freely today during our discussions.
- Everyone’s opinions are valuable.
- Please respect the opinions of each other.
- You don’t need to contribute to a specific question if you don’t feel comfortable.
- Please avoid sharing sensitive/personal information about specific patients.

Does anyone have any questions or queries before we start?

**[Participant introductions]:**

We’ll now go around, and I’d like everyone to have turns to introduce yourself. If you could tell us your name, how long have you been practicing podiatry, where in Aotearoa New Zealand do you practice and what your typical case load looks like (i.e., general care, sports, paediatrics, etc).

**[Opening questions]:**

1. How often do you come across 1st MTPJ injuries in your practice?

*• Prompts: For example, how many cases would you come across each week, month or year, or what proportion of your case load would have 1MTPJ injuries?*

1. How often are these 1^st^ MTPJ injuries diagnosed as sesamoiditis?
2. How would you define sesamoiditis?
3. Is sesamoiditis something you tend to diagnose provisionally or differentially in your practice?

***[PART A Key Questions related to assessment approaches]:***

1. What assessments do you perform to assist you with diagnosing sesamoiditis?

• *Prompts: palpation, observations, subjective history, imaging, range of motion tests, strength testing, pressure analysis, gait analysis, outcome measures, pain scales…*

1. How do you interpret the findings of these assessments to reach a diagnosis?
2. Is there any assessment(s) that you think is most helpful in diagnosing sesamoiditis?
3. Are there any other conditions that you are trying to rule out when performing these assessments?

• *Prompts: turf toe, gout flare…*

5. Is there anything else you use to assist you in reaching a diagnosis?

*• Prompts: specific subjective information from the patient (i.e., mechanism of injury), referral for imaging, discussion with a colleague....*

***[PART B Key Questions related to management approaches]:***

1. How do you usually manage a patient with sesamoiditis – what treatment tools do you use in the short-term?

• *Prompts: Offloading? Taping? Orthotics? Strength training? Stretches? Needing? Braces? Splints? Footwear? Insole modifications (felt or materials)? Moonboot/Square shoe/Forefoot offloading shoe? Massaging/soft tissue work? Avoidance of certain activities? Activity modification? Shockwave or radial therapy? Education?*

1. What treatment tools do you use in the long-term management?

*• Prompts: as above*

1. What influences your decision to opt for a specific treatment tool?

• *Prompts: For example, what characteristics/qualities would qualify for you to make the decision to use orthotic therapy for one patient and a moon boot for another?*

1. In your opinion, which treatment tool have you had most success with when managing a sesamoiditis case?

• *Prompts: Do you have any working theories on how this may help your patients?*

1. If you use orthotic therapy, can you describe the modifications and materials you use?

*• Prompts: forefoot modifications (Morton’s extension, cluffy extension, kinetic wedge, reverse ‘c’ plantar pad, ‘u’ cut out plantar pad, plantar pad, MT dome, MT bar), midfoot (arch fill), rearfoot (varus, valgus, heel rise, heel cushion) …*

• *Prompts: shell materials (semi-custom, function orthoses), poron (slow release, standard), EVA (220, 330, 350, etc), multiform, other foams, 3D, polypropylene, LDPE…*

1. At what point would you refer a patient to for more invasive/non-conservative treatment options?

*• Prompts: after 3 months, 6 months, etc, of no improvement in symptoms?*

***[Concluding Question]:***

Thank you all for your valuable input. This discussion has been very insightful. Just to summarise the main points from our discussion … [researcher to summarise discussion points].

To finish off, I’d like us all to have a turn at sharing any final comments on what you thought was the most important points from this discussion, or if there is anything else you would like to add that we haven’t covered yet?

***[Additional prompting Questions/Phrases that may be used]:***

- Could you tell us more about this…?
- What prompted you to do this/take this approach…?
- What characteristics drew you to….”x” ….?
- Interesting…when you think back to this time…is there any features or qualities you identify?
- Thank you for your input, it’s great.
- How do you feel about…”x” ….?
- When you say….”x”. What is your thought process behind this statement?
- In your opinion…would you say this is a challenge or an advantage?
- When do you seek a second opinion or further investigations/referrals?
- What resources did you wish you had access to?
- What qualities limited you…
- What features enabled you…
